# Evidence-based moisturizer selection for atopic dermatitis

**DOI:** 10.1016/j.jdin.2025.02.007

**Published:** 2025-05-17

**Authors:** Alain Taïeb, Marie Loden, Peter Schmid-Grendelmeir

**Affiliations:** a University of Bordeaux and INSERM U 1312, Bordeaux, France; b University of Uppsala and Eviderm Institute AB, Solna, Sweden; c Department of Dermatology, University of Zurich, Zurich, Switzerland

**Keywords:** atopic dermatitis, dysbiosis, emollients, moisturizers, pH, skin barrier

*To the Editor*: We read with interest the narrative review by Shobnam et al discussing behavioral interventions for atopic dermatitis (AD).^1^ The objective of the article was to “summarize the evidence base for these interventions” and to promote awareness and aid patients and caregivers. However, in contrast to systematic reviews, narrative reviews suffer from a lack of explicit methodology, which makes them less useful to identify suitable interventions, whereas gaps in the literature can be identified. At worst, misleading information may worsen the disease and undermine the vulnerable AD patients’ trust in caregivers. Such mistrusts are also more pronounced in areas where democratic institutions are less well-developed.

Therefore, the International Society for AD recently applied to include registered moisturizer creams in the 2025 revision of the World Health Organization essential medicines list to improve clinical outcome and reduce treatment costs in low-resource settings, where the access and affordability of appropriate moisturizers for AD are inadequate.^2^ The cost is prohibitive for patients belonging to the low to lower-middle income groups, where treatment costs for an international cream may well exceed the patients’ salary without being beneficial.

Shobham et al recommend treatments which belong to the lowest level in the evidence hierarchy (Figure 1), such as *in vitro* data of the so far poorly validated dysbiosis criterion,^1^^,^^3^ whereas established pharmacopeial standards to guarantee the microbial safety of moisturizers, skin pH, and barrier function (Figure 2) in AD are not even mentioned. They suggest differences in clinical efficacy based on nonvalidated *in vitro* antimicrobial activities, which instead may add “fuel to the fire” as emphasized by leading experts when discussing interventions on skin microbiote.^3^ Furthermore, we are surprised by the recommendation of ceramides for barrier improvement, based on the findings of a systematic review including 5 small studies, 3 of which reporting that transepidermal water loss values were not significantly different in subjects treated with ceramide-containing moisturizer versus controls and 2 of which finding a slight SCORing Atopic Dermatitis Index (SCORAD) improvement in the ceramide-treated group.^4^

**Fig 1:**
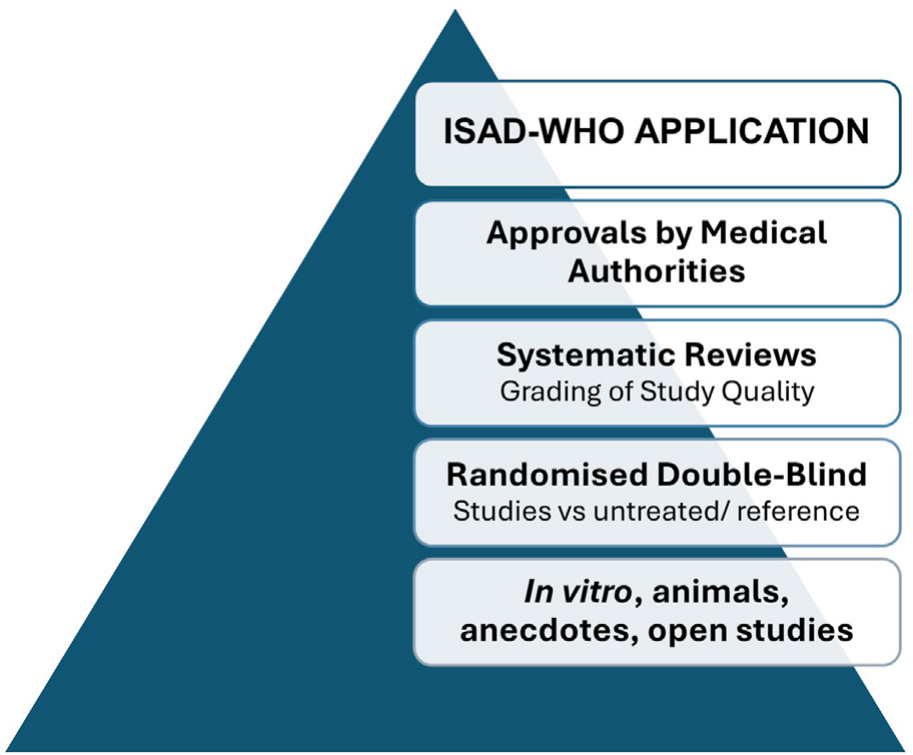
The hierarchy of evidence in our selection of moisturizers.

**Fig 2:**
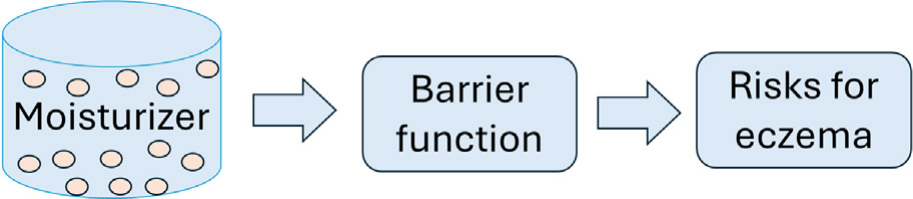
The risks for eczema are related to the changes in barrier function, where ingredients linked to improvement are recommended, whereas those with weakening effects are banned in the EML application, unless proven to be beneficial in clinical studies of atopic dermatitis.^2^*EML,* Essential medicines list.

Therefore, the essential medicines list proposal is based on systematic reviews of the efficacy of moisturizers in clinical studies in AD patients,^2^^,^^5^ along with, for example, pathophysiology guidance for the choice of established and authorized compositions for the treatment of AD by regulatory authorities (Figure 1). Furthermore, our recommendations adhere to those from, for example, the EU Scientific Committee of Consumer Safety with respect to selection of preservatives. Following consultations with manufacturing companies, our final selection was based on a consensus of the writing group∗ and endorsement by supporting organizations.∗∗

Hence, we recommend as the primary moisturizer 5% urea (carbamide), which delays relapses of AD, and as therapeutic alternatives 2 glycerol-containing creams (15% and 20%). Based on the estimated consumption and the manufacturing costs, the annual treatment cost is only €25-50 per patient.

In conclusion, the identification of moisturizers for AD should preferably be based on a hierarchy of data from authorized clinical research, while also addressing cost-efficiency and safety in low-resource settings.^2^

## Conflicts of interest

None disclosed.

## Footnotes

∗ Alain Taïeb (Dermatologist, ISAD France, Chair), Marie Loden (Pharmacist and Dermatologist, Eviderm Institute AB Sweden, Cochair), Peter Schmid-Grendelmeier (Dermatologist, ISAD Switzerland), Andreas Wollenberg (Dermatologist, ISAD Germany), Erere Otrofanowei (Dermatologist, ASDV, ISAD Nigeria), Ousmane Faye (Dermatologist, ISAD Mali), Gianni Baratto (Pharmacist, UNIFARCO, Italy), Andrea Baratto (Pharmacist, UNIFARCO, Italy), Roberta Milani (Pharmacist, UNIFARCO, Italy), Sophie Weber (Engineer, NAOS, France).

∗∗ ASDV (African Society of Dermatology and Venereology); ILDS (International League of Dermatological Societies); Global Skin (International alliance of Dermatology patients’ organizations).
